# Multilayer network analysis: new opportunities and challenges for studying animal social systems

**DOI:** 10.1093/cz/zoab006

**Published:** 2021-01-30

**Authors:** Matthew J Hasenjager, Matthew Silk, David N Fisher

**Affiliations:** 1 Department of Biological Sciences, Royal Holloway, University of London, Egham, UK; 2 Centre for Ecology and Conservation, University of Exeter, Exeter, UK; 3 School of Biological Sciences, University of Aberdeen, Aberdeen, UK

## Introduction

Many behavioral, ecological, and evolutionary processes are closely intertwined with patterns of social interactions, such as the evolution of cooperation (Croft et al. 2006), information and disease transmission (VanderWaal et al. 2014; Aplin et al. 2015), predator–prey dynamics (Ioannou et al. 2012), and dispersal decisions (Blumstein et al. 2009). Even in species where individuals are traditionally viewed as leading a relatively solitary existence, interactions occur across diverse contexts, including territorial defense, resource competition, and courtship. Moreover, among members of a population, there is often substantial variation in terms of whom individuals interact with, how frequently they do so, and the intensity of these interactions. Quantifying these patterns and elucidating their functional and ultimate consequences is a central goal of behavioral ecology (Whitehead 2009).

In recent years, these efforts have been facilitated by the widespread adoption of social network techniques imported from the physical and social sciences (Croft et al. 2008; Hasenjager and Dugatkin 2015; Krause et al. 2015). Network analysis provides a flexible framework for describing systems of interacting agents. In the context of animal populations, network nodes generally represent individuals, whereas connections between nodes (referred to as edges) quantify some form of social interaction, association, or relationship (e.g., agonistic, affiliative, proximity). Such networks are formally represented as an adjacency matrix or edge list, enabling the use of a rich set of mathematical tools for describing various aspects of a network’s structure (Whitehead 2009; Farine and Whitehead 2015). For instance, measures derived from networks can be used to characterize an individual’s influence over others (Flack et al. 2006; Rosenthal et al. 2015), the existence of subgroups within the population (Mersch et al. 2013), or how social relationships are structured according to phenotype (Aplin et al. 2013). In addition, these measures can facilitate investigation of the ecological and evolutionary consequences of social structure. For example, an individual’s position in a network can influence the speed it learns a new skill (Claidière et al. 2013), while network structure can influence how quickly a disease spreads through a population (Otterstatter and Thomson 2007).

Despite their flexibility, standard network approaches are not without limitations. Studies of animal social networks have traditionally represented social structure within a population using a network in which all edges represent the same type of relationship. For example, a network might quantify grooming interactions, spatiotemporal co-occurrences, or shared group membership. Yet animals can interact in different ways (e.g., grooming, play, aggression) and across different contexts (e.g., courtship, foraging). Considering only a single interaction type or combining multiple behaviors to produce a single aggregate measure may obscure important information about social structure (Finn et al. 2019). Furthermore, where multiple network types are considered (e.g., agonistic, affiliative), these are often analyzed independently of one another, tantamount to assuming that the patterning of each interaction type does not depend on the other(s). However, we know that this is unlikely to be the case in reality; agonistic interactions will change patterns of affiliative interactions not only among the interactants, but also their interactions with other group members and affiliative interactions among group members more widely. Social interactions can also be shaped by nonsocial forms of relationship, such as genetic relatedness or shared space-use, though incorporating such information using standard network approaches is not always straightforward (Pinter-Wollman et al. 2014). In addition, most network analyses use static network representations that provide “snapshots” of social structure at a particular point in time, whereas in reality, patterns of social interaction are dynamic, shifting in response to factors such as resource distributions, seasonal change, predation pressure, or demography (Blonder et al. 2012).

Multilayer network analysis has recently been proposed as a framework that can help to address these shortcomings (Silk et al. 2018; Finn et al. 2019). In brief, a multilayer network incorporates multiple sets of relationships into the same mathematical structure, often with each layer representing a distinct form of connectedness (e.g., a layer of grooming interactions and a layer of aggressive interactions, or layers for associations in different seasons). Crucially, because a multilayer formulation includes these networks within a single structure, the interdependencies between different forms of connectedness can be explicitly modeled and investigated. For example, an individual’s social importance may only become apparent when multiple forms of interactions are simultaneously considered (De Domenico et al. 2015; Beisner et al. 2020). Furthermore, layers are not limited to simply capturing different types of social interaction, but can also represent nonsocial forms of relationship (e.g., genetic relatedness, patterns of shared space-use), include different types of entities (e.g., nodes may represent physical locations in one layer and individuals in another), or represent different time points. By enabling the construction of more nuanced representations of social structure, multilayer approaches hold great potential to advance the study of animal social behavior and its relationship to ecological and evolutionary processes (Silk et al. 2018; Finn et al. 2019; Montiglio et al. 2020; Mourier et al. 2020).

For this Special Column, we have 2 primary aims. First, although a number of useful reviews have recently highlighted the potential of multilayer networks and related approaches for investigating animal behavior (Silk et al. 2018; Finn et al. 2019; Montiglio et al. 2020), there remain relatively few empirical studies that have employed these approaches thus far. The contributions to this Special Column help to fill this gap by applying multilayer network analysis to probe the causes and consequences of social structure across a diverse array of study systems. Second, as multilayer network analysis is still relatively new, there remains scant guidance on how best to employ these techniques. Multilayer networks inherit all the complexities of standard network analysis (see Farine and Whitehead 2015), while adding their own set of unique challenges (Finn et al. 2019). The contributions to this Special Column provide a wealth of practical guidance for researchers interested in employing these approaches, either serving as empirical case studies or explicitly addressing methodological questions. Here, we showcase how these contributions illustrate both the promise of multilayer networks and the challenges associated with their use.

## The Promise of Multilayer Network Analysis

In most social systems, not only do individuals engage in different forms of interaction, but these interactions often feedback on and influence one another. Multilayer network analysis offers a promising means to capture these complexities and to investigate how changes in 1 layer may drive dynamics in others. Bonnell et al. (2021) present a new method for investigating these sorts of dependencies within multilayer networks and illustrate its use by applying it to better understand power dynamics within vervet monkey *Chlorocebus pygerythrus* groups. First, a multilayer network was constructed with separate layers for grooming and aggressive interactions and further partitioned into separate male and female layers. This structure was then shifted through time to generate a time series of multilayer networks. Using a multivariate multilevel autoregression (MMAR) model, the authors found that changes in male–female grooming relationships cascaded throughout the network, driving changes in male dominance rank and male–male aggression, which in turn shaped future male–female grooming. The authors go on to illustrate how their MMAR approach can be extended in order to predict behavioral responses to social perturbations.

Beyond direct social interactions, individuals’ relationships can be described by various nonsocial dyadic measures, such as home-range overlap, differences in age, and the degree of genetic relatedness. Such pairwise measures can be represented in matrix form and therefore be incorporated into a multilayer network analysis. Equipped with long-term, high-resolution data on genetic relatedness and social associations within a free-ranging population of house mice *Mus musculus domesticus*, Evans et al. (2021) used multilayer network techniques to investigate the relationship between genetic relatedness and social structure within and across years. As individuals should avoid mating with relatives, the authors predicted that the genetic and social layers should overlap less in the breeding season, and this was indeed what they observed. The authors also observed reduced overlap between the genetic and social layers as population density increased, suggesting that as the intensity of resource competition increased individuals modified their interaction patterns to avoid associating with relatives. The approaches used by Evans et al. (2021) are broadly applicable and provide a promising means to investigate the links between social interactions and genetic relatedness.

As illustrated by the work of Evans et al. (2021), social interaction patterns are dynamic, shifting in response to internal and external influences. Temporal changes in social structure have typically been analyzed using extensions of monolayer network approaches (Blonder et al. 2012; Farine 2018), but it is also possible to represent each time point in a dynamic network as a layer in a multilayer network. This representation allows one to use the tools of multilayer network analysis to answer questions about temporal dynamics. Fisher and Pinter-Wollman (2021) take this approach to investigate how contact networks of a social spider, *Stegodyphus dumicola*, gradually change over time and relate changes in network structure to individual and collective behavior. By testing whether different temporal layers captured similar interaction patterns, they were able to identify the timescale over which spider networks changed. Their analysis further revealed that, despite dynamic changes in contact patterns, bolder spiders were consistently “keystone” individuals, characterized by a large and stable number of contacts. In providing a quantitative assessment of network stability, the reducibility analysis used by the authors could prove useful in many other study systems—for example, to compare social stability under different socio-ecological conditions or as a means to identify when societies undergo fundamental change.

## Challenges of Multilayer Network Analysis

Social network analysis is rarely straightforward, even when using monolayer networks. Researchers must think carefully about how to construct and analyze their network(s); what it represents; which metrics capture the biological phenomenon of interest; how to appropriately conduct hypothesis testing; the potential impact of missing or incorrect data, etc. (James et al. 2009; Whitehead 2009; Farine and Whitehead 2015; Silk et al. 2015; Farine 2017; Evans et al. 2020). Such considerations are at least as important for multilayer networks, given their added complexity. In addition, there are further considerations that are unique to multilayer networks. Which network layers should be included in an analysis? How should intra- and interlayer edges values be assigned? Is a particular network measure interpretable when edges in different layers represent dramatically different relationships? The contributions to this Special Column provide numerous examples of the decision-making processes involved in the effective application of multilayer network analysis.

Finn (2021) provides a primer on how multilayer networks may be used to investigate social systems by dissecting a multilayer structure into its component pieces and illustrating how each may provide unique insights into the structure and social dynamics of animal groups. For example, quantifying the degree of coupling between different network layers (e.g., aggression, affiliation) may offer novel insights into how different behaviors are functionally related and reveal variation in the strategies employed by individuals to cope with social challenges. They further identify common pitfalls associated with multilayer approaches and provide guidance on how these may be avoided. Throughout, Finn (2021) emphasizes the necessity for researchers to think carefully about how to build a multilayer network, what that network represents in terms of a species or group’s social ecology, and how to extract meaningful measures of social structure from that representation.

Although a key strength of multilayer network analysis is the ability to incorporate information on different forms of relationship, it may not always be appropriate to include all possible layers in an analysis (De Domenico et al. 2015; Finn et al. 2019). For instance, if layers reflect similar behavioral processes (e.g., 2 forms of threat display), they may contain redundant information, making it potentially desirable to either combine these layers or exclude 1 from the analysis. Yet guidance about how best to decide which layers to include in a multilayer analysis remains scarce. In the current issue, van der Marel et al. (2021) collected data on monk parakeets *Myiopsitta monachus* engaging in 2 types of directed agonistic interaction: direct displacements and noncontact aggression. Using simulations, the authors show that networks based on either behavior alone initially appear quite different, suggesting that they should not be aggregated into a single network layer. However, further simulations revealed this to be due to the relative frequency of the behaviors; direct displacements were much more commonly observed than noncontact aggression. After accounting for this difference, it was shown that these behaviors conveyed broadly similar information about parakeet social structure. Their approach promises to be broadly applicable for helping to decide which layers can be aggregated in a multilayer analysis.

As with monolayer networks, the results of a multilayer analysis can depend on how edge weights are assigned during network construction. Robitaille et al. (2021) used a multilayer approach to investigate how social associations varied across habitat types in a population of wild caribou *Rangifer tarandus* in Newfoundland. Each layer in the network corresponded to a different habitat type (lichen-rich foraging areas, forested regions, open habitats) with intralayer edges measuring association strength based on comembership in groups. Taking this approach required several decisions to be made, including the distance over which group membership should be defined and the spatial scale to use for land-use classification. To evaluate the sensitivity of their findings to these decisions, the authors tested a range of values for defining group membership and spatial scale, and then compared the properties of the resulting networks. Based on their analysis, the authors were able to identify appropriate social and spatial scales, dependent on their population and study objectives. For example, as foraging areas tended to be scarcer and more fragmented, social connectivity in that habitat layer was particularly sensitive to the spatial resolution used during habitat classification. Together, the authors’ findings demonstrate how conclusions derived from a multilayer analysis can crucially depend on the social, spatial, and temporal scales used in its construction and illustrate an approach for matching these scales to research objectives.

## The Future of Multilayer Networks

Contributions to this Special Column provide examples of the promise of multilayer networks in animal behavior, appropriately tempered with an appreciation for the complexities involved in their use. Yet the application of multilayer networks to investigate animal social systems is in its infancy and there remains great scope for researchers to use these approaches creatively to address novel questions about the ecology and evolution of social behavior. For instance, a multilayer formulation can be used to not only represent different relationships among the same set of individuals, but also interconnected systems where the nodes within each layer represent distinct types of entity (Kivelä et al. 2014; Finn et al. 2019). A layer representing the social networks of one or more animal populations could thus be embedded within a layer that represents physical locations within the environment and help to unify the analysis of spatial and social networks (Albery et al. 2021). Such a framework could prove useful in investigating how social interactions and the physical connectedness of the environment jointly shape dispersal patterns or responses to human-induced environmental change. Similarly, a multilayer framework could be used to model the embeddedness of networks of interacting genes, protein complexes, and physiological systems within individual organisms. Multilayer approaches thus have the potential to explicitly link different levels of biological organization and facilitate investigations of how interactions at 1 organizational level can effect changes in higher or lower levels (Cantor et al. 2017; Montiglio et al. 2020). By transcending scale in this way, multilayer networks can therefore provide a natural approach to integrate behavioral research within other fields such as disease ecology and community ecology. In the era of big data, where technology is allowing us to gather ever more detailed molecular, physiological, behavioral, and environmental data about our study systems (Krause et al. 2013; King et al. 2018), multilayer networks provide a flexible framework to help us visualize, analyze, and interpret this wealth of information.
